# Memory decay enhances central bias in time perception

**DOI:** 10.1177/20416695221140428

**Published:** 2022-12-05

**Authors:** Natsuki Ueda, Kanji Tanaka, Katsumi Watanabe

**Affiliations:** National Institute of Mental Health, 26353 National Center of Neurology and Psychiatry, Tokyo, Japan; Integrative Brain Imaging Center, 26353 National Center of Neurology and Psychiatry, Tokyo, Japan; Faculty of Arts and Science, Kyushu University, Japan; 13148 Faculty of Science and Engineering, Waseda University, Japan

**Keywords:** central bias, short-term memory, memory decay, retention curve, temporal expectation, perceptual inference, time perception

## Abstract

Temporal expectations are essential for appropriately interacting with the environment, but they can be biased. This tendency, called central bias, places higher weights on expected rather than actual duration distributions when perceiving incoming sensory stimuli. In particular, the central bias is strengthened in order to decrease total response error when incoming sensory stimuli are unclear. In the present study, we investigated whether the central bias was enhanced via memory decay. For this, we used a delayed reproduction task, manipulating retention periods by introducing delays between the sample interval and the reproduction phase (0.4, 2, 4 s in Experiment 1; 0.4, 2, 8 s in Experiments 2 and 3). Through three experiments, we found the gradual strengthening of the central bias as a function of the retention period (i.e., short-term memory decay). This suggests that the integration of temporal expectation, generated from past trials and stored sensory stimuli, in a current trial occurs in the reproduction phase in the delayed reproduction task.

## Introduction

Conceptually, central bias refers to gravitation toward the central value of presented intervals. For instance, people tend to overestimate shorter and underestimate longer intervals during interval reproduction tasks. In previous studies, central bias was reported in color (Olkkonen & Allred, 2014; Olkkonen et al., 2014), timing (Cicchini et al., 2012; Jazayeri & Shadlen, 2010; Karaminis et al., 2016; Murai & Yotsumoto, 2018), and line length tasks (Ashourian & Loewenstein, 2011). In addition, Roach et al. (2017) revealed that this bias was also induced across distinct sensory modalities (e.g., vision and audition), albeit it was represented separately if participants required distinct motor responses (e.g., manual and vocal reproduction). Accordingly, central bias seems to be ubiquitous in perceptual judgment processes and may reduce the variance of reproduction times in an ambiguous environment.

Multiple information processing models have been proposed for time perception (Gibbon et al., 1984; Shi et al., 2013). According to Jazayeri and Shadlen (2010), a Bayesian strategy model for interval reproduction comprised three stages (see also a similar three-step model proposed in scalar expectancy theory; Buhusi & Meck, 2005; Coull et al., 2016): the first stage outlines the measurement process of current sensory information (i.e., likelihood); in the second stage, Bayesian inference is computed by combining likelihood with prior knowledge of the world (i.e., prior); in the third stage, the estimated sample interval is converted into production times. The Bayesian framework for perceptual inference highlights that central bias is expressed by the representation of a mean stimulus set and optimal integration of the incoming sensory stimuli (Jazayeri & Shadlen, 2010; Karaminis et al., 2016; Roach et al., 2017). For example, when the participants’ sensory resolution was low (i.e., a process in the first stage), their reproduction tended to be more biased to reduce total response error (Cicchini et al., 2012).

Importantly, the reliability of incoming sensory stimuli is influenced by both internal and external noises. Internal noise is instigated by memory retention, while external noise is generated by stimulus noise. For example, Olkkonen et al. (2014) used a delayed hue comparison task to demonstrate that internal (i.e., memory retention; the delay between the reference and estimate) and external noise (i.e., chromatic noise) strengthen the central bias in color perception. Participants in their study were asked to evaluate two color stimuli and determine which stimulus was yellower (or bluer), and the findings showed that the central bias was strengthened owing to the increase in internal and external noises.

The relationship between memory and time perception has also been investigated (Droit-Volet et al., 2007; Jones & Wearden, 2003; Taatgen & van Rijn, 2011; Takahashi & Watanabe, 2012; Wearden et al., 2002). First, Wearden et al. (2002) used an interval comparison task in which participants learned a first interval (the standard intervals were randomly chosen between 400 and 600 ms), which was retained in the short-term memory (1, 2, 5, or 10 s); thereafter, a second interval was presented for comparison with the standard interval. The results showed that short-term memory decay disrupts interval judgment (i.e., increasing of response variability). Second, Rattat and Droit-Volet (2010) noted no effect of retention periods on interval judgment, and that the effect of memory retention on reference memory might not affect temporal judgment. In their study, participants learned a standard interval (4 s) during the learning phase. During the testing phase, the comparison interval was presented, and participants were asked to select whether the stimulus interval was the same as the standard interval shown immediately after the learning phase (i.e., 15 min after) or the one presented 24 h later. They found that the retention periods had no significant effect on temporal judgment. Third, Roach et al. (2017) showed that perceptual priors converge toward a common central duration when the experimental block interleaved two stimuli distributions. The converged distribution may be stored in the reference memory, and then affect decision-making in the time perception process as a central bias. Fourth, a previous study using a computational approach showed that patients with mild cognitive impairment (i.e., considered as the precursor of Alzheimer's disease and dementia) had a stronger central bias than healthy participants and that this was due to the combination of a narrower prior and perceptual unreliability (Maaß et al., 2021). Hence, the influencing factors of central bias may be related to not only perceptual reliability but also brain function alteration.

In sum, the current literature shows that participants’ reproduction may be impacted by two independent variables: the effect of reference memory and memory decay in short-term memory (which can be manipulated by retention period). Still, although temporal judgment may not be affected by reference memory (e.g., Rattat & Droit-Volet, 2010), it may be affected by short-term memory. Thus, we can assume that once people learn a certain interval, they retain it in the short-term memory module and the interval is influenced by the previously learned interval in their reference memory. However, the effect of short-term memory decay on perceptual inference remains unknown; specifically, investigations are lacking as to whether short-term memory decay changes central bias as a result of perceptual inference or whether it simply reduces the precision of participants’ reproduction and does not modulate central bias. For example, although Droit-Volet et al. (2007) demonstrated that a longer retention period deteriorated perceptual precision in an interval comparison task, these authors did not examine the relationship between low precision and central bias.

In the present study, we investigated whether short-term memory decay would affect perceptual inference. Across the three experiments, we used the modified version of the interval reproduction task proposed by Roach et al. (2017) and Ueda et al. (2022) wherein participants reproduce the interval of the presented visual stimulus, to test the effect of short-term memory retention on central bias in terms of time perception (we named it delayed interval reproduction task). The stimulus had seven interval variations, a pseudorandom order of visual stimuli, and the stimulus interval was subsequently reproduced in the interval after the delay. We assumed that participants learned the stimulus distribution during each experimental block and stored the statistical regularity in the reference memory.

To manipulate the effect of memory decay, each trial had a retention period between the reference and reproduction. If the retention period is increased, the uncertainty of the retained perceptual information will be reflected in the reproduction phase, prior expectation stored in reference memory will be dominated by short-term memory decay, which will then strengthen the central bias. Another possibility is that participants make their decision immediately after the presentation of the stimulus interval (i.e., the first and second stages occur immediately after one another or even simultaneously), and that they simply retain their temporal judgement. A neurophysiological study that used the interval reproduction task showed that effect of perceptual bias can already be seen in the perceptual phase, indicating that prior knowledge may already influence perceptual judgement in some way during stimulus presentation (Damsma et al., 2021). If participants make perceptual judgements immediately after or simultaneously with stimulus presentation, the central bias would not be strengthened by memory decay, and only the variability of temporal reproduction would increase.

## Experiment 1

### Materials and Methods

#### Participants

In total, 36 people participated (age range: 18–27 years) and provided written informed consent. All participants had normal or corrected-to-normal visual acuity. This experiment was approved by the Institutional Review Board of Waseda University and the National Center of Neurology and Psychiatry.

#### Apparatus and Stimuli

Stimuli were displayed on a 23-inch LCD monitor (60 Hz frame rate). Visual stimuli constituted isotropic Gaussian patches (sigma = 10) generated in MATLAB using Psychtoolbox for MATLAB (Brainard, 1997; Kleiner et al., 2007; Pelli, 1997) on a gray background. Participants viewed the display from a 60 cm distance in a dimly lit setting controlled by a chin rest.

#### Task Procedure

**Figure 1.**
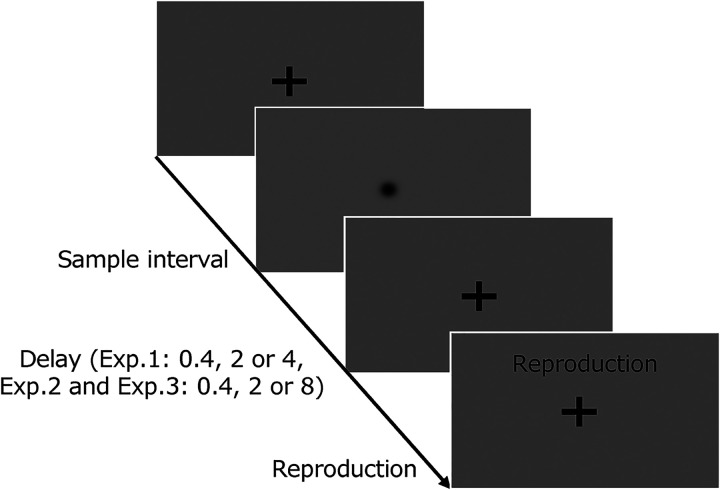
Experimental task in experiment 1. The sequence of events during a trial. The appearance of a fixation cross signaled the initiation of a trial. Participants were instructed to fixate on the cross and maintain it throughout the trial. After a random delay (0.5–1.2 s), a visual disk was displayed for a specific interval. Participants were then instructed to estimate the sample interval and reproduce it by pressing a button when the word “reproduction” appeared on the screen.

Each trial commenced with a fixation cross, and participants were instructed to fixate on the cross and maintain the fixation throughout the trial. After the presentation of the fixation cross with a random delay (0.5–1.2 s), a visual disk (isotropic Gaussian patch) was presented for a certain interval. Following the methods in the studies by Roach et al. (2017) and Ueda et al. (2022), the interval distributions comprised seven types centered at 640 ms, with a step size of 0.15 log units in each block (approximately 227, 430, 453, 640, 904, 1276, and 1803 ms). After a certain delay (0.4, 2, or 4 s), participants were required to reproduce the interval by holding down the space key on the keyboard for the appropriate time (Figure 1).

Following Olkkonen et al. (2014), the task had three delay conditions: short, intermediate, and long. The given delay between the target and reproduction was 0.4 s in the short condition, 2 s in the intermediate condition, and 4 s in the long condition. The time between the key press and key release was defined as the behavioral measure of “reproduced duration,” and the period wherein the visual disk was shown was assigned as the “interval.” No feedback was provided in the trials, and participants were not informed about interval distribution. The participants completed 70 trials in one block for each delay condition, totalizing 210 trials. In each delay condition, each of the seven types of intervals was presented 10 times in a pseudorandom order. The order of the blocks was randomized between participants.

#### Data Analysis

All analyses were implemented using R. To achieve normal distributions of the data in Experiments 1, 2, and 3 both the stimuli intervals and reproduction durations data were logarithmically transformed across the whole data analysis process.

#### Linear Mixed Model

To determine whether the prior distribution impacted the strength of the central bias, we fitted each dataset with a linear mixed-effect model (LMM)—using lme4 library version 1.1.15 (Bates et al., 2015) and lmerTest library version 3.1-3 to calculate the *p*-value (Kuznetsova et al., 2017)—in R (R Core Team., 2016).

We fitted the LMM with reproduced duration as the outcome variable, the sample intervals, block, and interaction between sample interval and block as the fixed effects, and the participants as the random effect. In the LMM, the estimation of the coefficient for the sample interval virtually corresponded to a regression slope of the reproduced duration against the sample interval; hence, we called this coefficient the “slope” hereafter. A slope value smaller than 1 indicates the occurrence of central bias, and even smaller values indicate a stronger central bias.

We evaluated the relationship between interval and duration by fitting the linear model *y* *=* *ax* *+* *b* for each block of each participant, and the indifference point was calculated following the methodology proposed in two prior studies (Roach et al., 2017; Ueda et al., 2022). This serves to show the intersection point between the fitted line from the model and a unity line representing veridical performance. Equation (1) is as follows:

(1) $$\begin{array}{c} I\;=\;\frac{b}{1-a}. \end{array}$$

where *I* is the point of indifference, *a* is the slope (correlation coefficient), and *b* is the intercept of the linear model in Equation (1). The indifference point depicts the center of gravity of the duration. Therefore, if the indifference point varies across conditions, this means that the participants’ reproduction is affected by extraneous variables such as the subjective shortening effect, wherein the subjective timing is shortened via memory retention (Droit-Volet et al., 2007).

We compared the indifference points (*I*) across conditions using LMM because our data might include variations in subjective timing by the participant. For comparing indifference points, we included indifference point as the outcome variable, condition as the fixed effect, and participants as the random effect.

#### Accuracy and Precision

Following previous studies (Cicchini et al., 2012; Karaminis et al., 2016), the total error in the production task was segmented into two parts: one reflecting a systematic offset from real values (*VE*) and the other reflecting the scatter around the mean (coefficient of variation, *CV*). Smaller *VE* values indicate small differences between the interval and the participants’ reproduced duration, so it was used as an accuracy parameter; meanwhile, smaller *CV* values indicate less variability in participants’ reproduction, so it was utilized as a precision parameter (Karaminis et al., 2016). We subtracted the mean reproduction time *R* from each reproduced time $R_{i,n}$ (where *i* stands for the stimulus interval and *n* indicates the repetition throughout the experiment), and then added the average stimulus interval *S* as follows:

(2) $$\begin{array}{c} {R^{\prime }}_{i,n\;}=\;R_{i,n}\;-\;\bar{R}+\;\bar{S.} \end{array}$$

For a given stimulus interval *i*, $VE_{i}$ is the normalized difference between the average produced time and a certain stimulus interval.

(3) $$\begin{array}{c} VE_{i}\;=\;\frac{\left|\bar{R_{i}^{\prime }}-\;S_{i}\right|}{\bar{S}}. \end{array}$$

*CV* is given by the standard deviation of the data points for each duration, as normalized by mean duration.

(4) $$\begin{array}{c} CV_{i}\;=\;\frac{\sqrt{\frac{\sum {\left(R_{i}^{\prime }\;-\;\bar{R_{i}^{\prime }}\right)}^{2}}{N}}}{\bar{S}}. \end{array}$$

The *CV* and *VE* were compared across conditions using LMM. To test the differences by each condition regrading *CV* and *VE*, we ran different LMM models with *CV* and *VE* as outcome variables, with condition as the fixed effect, and participants as the random effect.

### Results and Discussion

**Figure 2.**
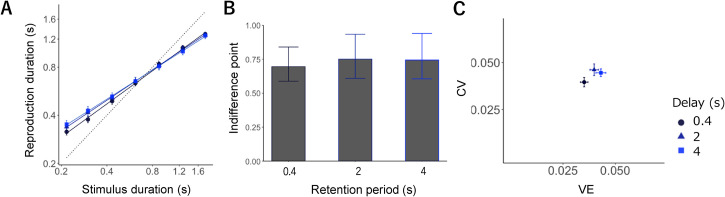
(A) mean reproduction durations in separate testing blocks. Error bars indicate standard error (*SE*). Each symbol expresses a retention period: 0.4 s (circle, dark blue), 2 s (triangle, medium blue), and 4 s (rectangle, blue). (B) Indifference points showing the intersection point for the veridical line and the estimated regression line in each retention period. (C) CV (normalized average root variance of the reproductions) plotted against VE (difference between average production time and physical sample interval).

LMM analysis showed the significant effect of sample interval (*F* *=* 5223.26, *p* < .01), condition (*F* *=* 8.58, *p* < .01), and the interaction between interval and condition (*F* *=* 7.48, *p* < .01). The participants reproduced the duration in each condition, with short intervals biased toward longer intervals and long intervals biased toward shorter intervals; the estimated slopes (i.e., regression coefficients) in each condition were as follows: 0.67 for the short, 0.61 for the intermediate, and 0.59 for the long condition, with lower slope values implying a stronger central bias (refer to Figure 2A).

We tested slope differences between each two conditions (i.e., short vs. intermediate, intermediate vs. long, and short vs. long) to compare the degree of delay-induced bias. We found significant differences, with the slope in the short condition being larger than in the intermediate (*t* *=* 2.66*, p* = .01) and long conditions (*t* *=* 3.76*, p* < .01). Nonetheless, there was no evident and significant difference between the slopes in the intermediate and long conditions (*t* *=* 1.09*, p* = .27, all *p-values* were Holm-adjusted).

LMM was then applied to investigate the condition-effect of contrast in the indifference point, showing the intersection of the veridical performance and slope between the three conditions. No significant effect of condition was discovered (*F* = 0.34, *p* = .71), indicating that all conditions had similar gravitation points of reproduced duration, as well as that the delay seemed to strengthen the central bias between the short and intermediate conditions, without altering the gravitation point of duration across conditions (Figure 2B).

Figure 2C illustrates the average *CV* against the *VE*. The LMM results for *CV* presented a significant effect for condition (*F* = 3.54, *p* = .03), and multiple comparisons demonstrated that the *CV* of the short was smaller than that of the intermediate condition (*t* = 2.54, *p* = .03); nonetheless, the differences between the intermediate and long conditions (*t* = 0.63, *p* = .53) and between the short and long conditions (*t* = 1.91, *p* = .12) were not significant (all *p-values* were Holm-adjusted).

The LMM results for *VE* also displayed a significant effect for condition (*F* = 7.62, *p* < .01), and multiple comparisons showed that the *VE* for the short condition was significantly smaller than that for the intermediate (*t* = 2.32, *p* = .04) and long conditions (*t* = 4.88, *p* < .01). Nevertheless, the difference between the intermediate and long conditions was not significant (*t* = 1.56, *p* = .12; all *p-values* were Holm-adjusted). The outcomes for *VE* (i.e., absolute value of vertical error) were consistent with the slope analysis, with no significant difference between the intermediate and long conditions. The increase of central bias was accompanied by an increase in the *CV*, except for the long condition, where there was no increase in *CV* and an increase in *VE* and slope value.

In Experiment 1, we determined an association between the delay and strength of the central bias. There was seemingly a trend of a positive relationship between the retention period and central bias across participants; however, no significant differences were found between the intermediate and long conditions for slope, *VE*, and *CV*. There are two possible reasons for the lack of enhancement of the central bias between the 2 s and 4 s retention periods. One is that the association was limited to less than 2 s (i.e., the bias saturates after more than 2 s), and another is that the retention period between 2 s and 4 s was not long enough to make a difference on the strength of the central bias in time perception. Therefore, we sought to determine whether a longer period could also affect the strength of the bias in Experiment 2, wherein we extended the delay of the longest retention period: short (0.4 s), intermediate (2 s), and long (8 s).

## Experiment 2

### Materials and Methods

#### Participants

Altogether, 35 participants participated in Experiment 2 (age range: 18–35 years) and provided written informed consent. All participants had normal or corrected-to-normal visual acuity. This experiment was approved by the institutional review board of Waseda University and the National Center of Neurology and Psychiatry.

#### Stimuli and Procedure

Except for the change in the long retention period (8 s), the experimental methodology of Experiment 2 was identical to that of Experiment 1.

### Results and Discussion

LMM analysis showed the effect of sample interval (*F* *=* 5971.09, *p* < .001), condition (*F* *=* 18.95, *p* < .001), and the interaction between interval and condition (*F* *=* 18.06 *p* < .001). As in Experiment 1, the participants reproduced the duration, with short intervals biased toward the longer intervals and long intervals biased toward the shorter intervals; the slopes were 0.76, 0.70, and 0.63 for the short, intermediate, and long conditions, respectively.

**Figure 3.**
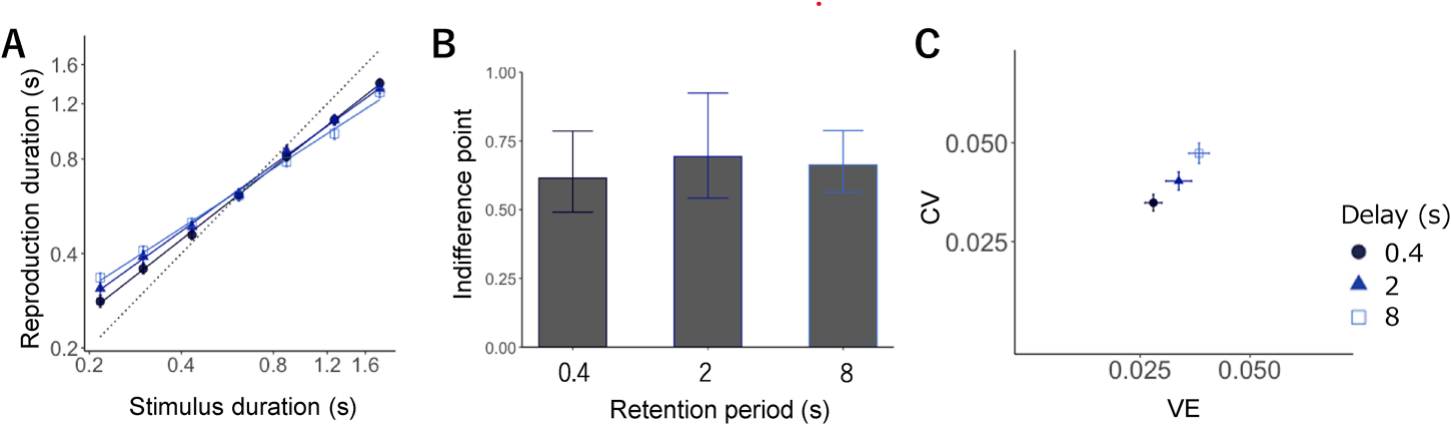
(A) Mean reproduction durations in separate testing blocks. Error bars display standard error (SE). Each symbol expresses a retention period: 0.4 s (circle, dark blue), 2 s (triangle, medium blue), and 8 s (rectangle, blue). (B) Indifference points showing the intersection point of the veridical line and the estimated regression line in each retention period. (C) CV (normalized average root variance of the reproductions) plotted against VE (difference between average production time and physical sample interval).

Then, slope differences were calculated for all conditions. The slope for the short condition was significantly larger than that for the intermediate (*t* = 2.75*, p* = .01) and long conditions (*t* = 6.00, *p* < .01), and that for the intermediate was significantly larger than that for the long condition (*t* = 3.24, *p* < .01, all *p-values* were Holm-adjusted; Figure 3A). These findings imply that the central bias was strengthened by a delay between the interval and reproduction.

We quantified the difference in indifference points between the three conditions through an LMM and found no significant effect of the condition (*F* = 0.21, *p* = .81). Although there was no significant difference in the gravitation point of reproduction in each condition, the strength of the central bias gradually increased due to delay (Figure 3B).

Figure 3C illustrates the average *CV* against *VE*. LMM revealed the significant main effect of the condition on *CV* (*F* = 14.93, *p* < .01) and *VE* (*F* = 18.31, *p* < .01). Multiple comparisons showed that the *CV* for the short condition was significantly smaller than that for the intermediate condition (*t* = 2.39, *p* = .02); that for the intermediate condition was significantly smaller than that for the long condition (*t* = 3.06, *p* < .01); that for the short condition was significantly smaller than that for the long condition (*t* = 5.45, *p* < .01, all *p-values* were Holm-adjusted).

Moreover, the short condition showed significantly smaller *VE* compared with the intermediate (*t* = 2.88, *p* = .01) and long conditions (*t* = 5.20, *p* < .01), and it was also smaller in the intermediate than in the long condition (*t* = 2.31, *p* = .02, all *p-values* were Holm-adjusted). That is, *VE* gradually increased with the delay between the interval and reproduction, accompanied by an increase in the *CV*. These tendencies were consistent with those in Experiment 1.

The main difference in the results of these two experiments was that there was no difference in central bias strength between the intermediate and long retention periods in Experiment 1 (2 s vs. 4 s), but there was an enhancement in such strength in Experiment 2 (2 s vs. 8 s). Previous studies proposed that perceptual variability increases due to a rise in the retention period of interval reproduction, as well as that such increase in the variability is unsteady or nonlinear (Hesse & Franz, 2010; Pearson et al., 2014; Rademaker et al., 2018). Therefore, the stepwise increase in Experiment 2 may be because the difference between the retention periods of 2 and 8 s was long enough to withstand the unsteady or nonlinear increase in sensory variability.

Although the results demonstrated that the central bias was strengthened as a function of the retention period, one concern remains to be solved. As the retention periods in Experiment 2 were fixed in each block, the participants might have decreased their attention (or vigilance) toward the task and its performance, including in terms of the stored memory. Therefore, it may be possible to interpret the present results in that the enhanced central bias was not due to memory decay, but to a decrease in attention during the retention period. To investigate this potential problem, in Experiment 3, we mixed the retention periods in each block to ensure that participants needed to pay attention to the task at hand.

## Experiment 3

### Materials and Methods

#### Participants

Altogether, 35 participants participated in Experiment 3 (age range: 18–24 years) and provided written informed consent. All participants had normal or corrected-to-normal visual acuity. This experiment was approved by the institutional review board of Kyushu University and the National Center of Neurology and Psychiatry. We excluded the data of one participant from the analysis due to showing a reaction time more than five times away from the sample interval on more than 30 trials.

#### Stimuli and Procedure

The stimuli and procedures of Experiment 3 were identical to those in Experiment 2, except that the three retention periods (0.4, 2, and 8 s) were mixed in each block.

### Results and Discussion

**Figure 4.**
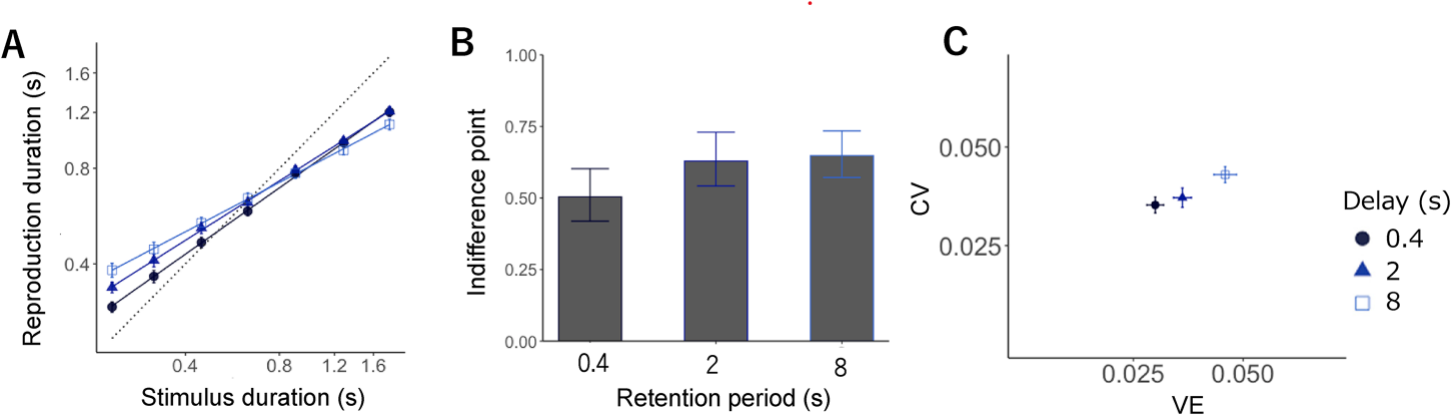
(A) Mean reproduction durations in separate testing blocks. Error bars display standard error (SE). Each symbol expresses a retention period: 0.4 s (circle, dark blue), 2 s (triangle, medium blue), and 8 s (rectangle, blue). (B) Indifference points showing the intersection point of the veridical line and the estimated regression line in each retention period. (C) CV (normalized average root variance of the reproductions) plotted against VE (difference between average production time and physical sample interval).

LMM analysis showed the effect of sample interval (*F* *=* 8220.65, *p* < .01), condition (*F* *=* 222.18, *p* < .01), and the interaction between interval and condition (*F* *=* 200.93, *p* < .01). As in Experiments 1 and 2, the participants reproduced the duration, with short intervals biased toward longer intervals and long intervals biased toward the shorter intervals; the slopes were 0.69, 0.62, and 0.51 for the short, intermediate, and long conditions, respectively. Significant slope differences were found across the three conditions, with the slope for the short condition being significantly larger than that for the intermediate (*t* = 4.31*, p* < .01) and long conditions (*t* = 10.64*, p* < .01); further, the slope for the intermediate condition was significantly larger than that for the long condition (*t* = 6.33, *p* < .01, all *p-values* were Holm-adjusted; Figure 4A). These findings imply that the central bias was strengthened by a delay between the interval and reproduction.

The LMM for the difference in indifference points across conditions demonstrated the significant effect of condition (*F* = 26.10, *p* < .01). A post-hoc analysis further depicted that the indifference point for the short condition was significantly smaller than that for the intermediate (*t* = 6.17, *p* < .01) and long conditions (*t* = 6.33, *p* < .01); still, the difference between the intermediate and long conditions was non-significant (*t* = 0.15, *p* = .87, all *p-values* were Holm-adjusted; Figure 4B).

LMM for *CV* revealed a significant main effect of condition on *CV* (*F* = 21.06, *p* < .01) and *VE* (*F* = 95.26, *p* < .01). Regarding *CV*, multiple comparisons failed to show differences between the short and intermediate conditions (*t* = 1.27, *p* = .20). Still, the *CV* for the intermediate condition was significantly smaller than that for the long condition (*t* = 4.06, *p* < .01), and that for the short condition was significantly smaller than that for the long condition (*t* = 5.34, *p* < .01, all *p-values* were Holm-adjusted). Regarding *VE*, it was significantly smaller for the short than the intermediate (*t* = 5.33, *p* < .01) and long conditions (*t* = 13.04, *p* < .01), and significantly smaller for the intermediate than the long condition (*t* = 7.70, *p* < .01; all *p-values* were Holm-adjusted).

Although we did not find an enhancement in central bias between the intermediate and long conditions in Experiment 1 (2 s vs. 4 s), we did find such an enhancement in Experiments 2 and 3 (2 s vs. 8 s). This difference might be due to the tendency for the central bias to be enhanced as the ambiguity of the sensory representation increases due to memory decay, which decays nonlinearly. As an additional exploratory analysis, we evaluated whether *CV* and *VE* increased linearly or logarithmically as a function of the retention period. The linear model fit *CV* better than the logarithmic model (i.e., the AIC values of the linear models were smaller than those of logarithm models), with the Δ *AIC* being 451.46, 469.49, and 469.30 for Experiments 1, 2, and 3, respectively. Regarding *VE*, the linear models fit better than the logarithmic model, with the Δ *AIC* being 535.07, 554.77, and 536.95 for Experiments 1, 2, and 3, respectively.

## General Discussion

In this study, we demonstrated that central bias in interval judgment was strengthened with memory decay. Specifically, although we found a difference in central bias only between the retention periods of 0.4 and 2 s in Experiment 1, Experiments 2 and 3 showed significant differences between the retention periods of 0.4 and 2 s and of 2 and 8 s. Further, despite the retention periods being mixed in the block in Experiment 3, the central bias results were similar to those in Experiment 2. The decrease in reliability of the sensory information due to the retention period was reflected in the strength of central bias, showing the result of the integration between the statistical characteristics of prior sensory information and the incoming sensory stimuli. In other words, perceptual decision making due to integration of sensory and prior information takes place around the reproduction phase in the interval reproduction task.

This study investigated whether short-term memory retention would impact perceptual inference processes. We hypothesized that if participants integrated the prior and likelihood in the reproduction phase, they would monitor the reliability of the measurement during the retention period; alternatively, if they integrated the prior and likelihood immediately after interval presentation, they would simply retain the estimation, and the central bias would not be strengthened by the retention period—instead, only the variability would increase. Our results verified that the strengthening of the central bias was attributed to the increase in retention period, suggesting that the degree of temporal reliability is updated during the retention period. Thus, in our delayed interval reproduction task, the perceptual inference was completed in the following order (and some processes probably occurred simultaneously): initially, participants measured the presented stimulus interval; next, they retained the measurement during the retention period; ultimately, the likelihood (i.e., which depends on the certainty of retained measurement) and prior information that was learned in the previous stimuli set for the current block was integrated, resulting in a central bias. Using a cued visual reaction time task, one previous study proved that the temporal prediction is definitively updated, is accompanied by temporal probability, and the update correlates with a brain region (Coull et al., 2016). Therefore, there is likely an update system for perceptual inference in time perception.

The results of Experiments 2 and 3 were not completely consistent. In Experiment 2, the strength of the central bias gradually increased from retention periods 0.4–8 s together with an increase in *CV*, and the indifference points across retention period conditions did not differ significantly. Contrary to this, in Experiment 3, the *CV* did not increase between retention periods 0.4 s and 2 s, albeit the central bias did show an increase in strength. Additionally, the indifference point for the retention period of 0.4 s was smaller than that for the other retention period conditions, indicating that participants generally underestimated the perceived interval in the trial for this retention period.

One possible explanation for these results is that the effect of retention time on perceptual inference is compounded by the effect of temporal unpredictability, which in turn modulates attention. That is, the temporal certainty between events (i.e., the predictability of when an event will occur) modulates attention and the perceptual threshold. For example, a study showed that in temporal discrimination tasks (i.e., participants are required to judge whether the presented interval is longer or shorter than the pre-learned interval), if participants do not know when the target interval will be presented, the longer the participant wait for the onset of the target interval, the higher the participants’ discrimination performance (Grondin & Rammsayer, 2003). This effect may be caused by the role that attention plays in temporal prediction, wherein one's attention gets devoted to temporal information as the waiting time for the perceptual event lengthens. Previous studies revealed that if attention is diverted away from a target interval, the subjective time is shortened and the variability of the interval estimation is increased (Polti et al., 2018). Therefore, the increase in *CV* and the underestimation of the interval in the retention period of 0.4 s in Experiment 3 may have been caused by the participants’ attention being diverted away from interval reproduction due to temporal unpredictability.

Our model comparisons showed that the linear model fitted the increase in *CV* and *VE* with retention period better than the logarithm model. Several studies have indicated that perceptual memory declines nonlinearly (Anderson, Campbell et al., 2014; Hesse & Franz, 2010). For example, Hesse and Franz (2010) observed that, at first, memory for visual information about the movement environment degraded rapidly, and went on to degrade more slowly as the retention periods increased. Meanwhile, Rademaker et al. (2018) showed that the visual working memory has a constant decrease. One perspective that may explain the differences in these studies regarding the increase in perceptual variability is the effect of initial states of perceptual discriminability due to task difficulty. For example, White (2001) indicated that if the initial state of perceptual discriminability is low, the nonlinear memory decay curve is steeper, and if it is high, the curve is gradual and approaches linearity.

This study has two major limitations. First, our delayed reproduction task has only one initial state condition, namely, only one interval distribution for three different retention periods; meanwhile, previous studies used multiple interval distributions (Cicchini et al., 2012; Jazayeri & Shadlen, 2010; Karaminis et al., 2016; Ueda et al., 2022). Second, only three retention periods were adopted (0.4, 2, and 4 s in Experiment 1; 0.4, 2, 8 s in Experiments 2 and 3), while more than four retention period conditions were used in previous studies (Rademaker et al., 2018; White, 2001). To provide more knowledge on the model that better fits the decay curve, future works could use more interval distribution types and retention periods.

In conclusion, an increase in the retention period strengthened the central bias in interval reproduction by increasing memory decay. These results show that the increased sensory variability due to a rise in retention period is reflected in the strength of the central bias in the participants’ reproduction, as well as suggest that perceptual decisions in interval reproduction tasks are made somewhere along the reproduction phase.
